# Chatbot Language - crowdsource perceptions and reactions to dialogue systems to inform dialogue design decisions

**DOI:** 10.3758/s13428-022-01864-x

**Published:** 2022-06-14

**Authors:** Birgit Popp, Philip Lalone, Anna Leschanowsky

**Affiliations:** Fraunhofer IIS, Am Wolfsmantel 33, 91058 Erlangen, Germany

**Keywords:** Chatbot, Conversational user interface, CUI, Dialogue system, Speech assistant, Dialogue strategy, Dialogue design, Survey, Simulation, Survey, JavaScript, Crowdsourcing, Crowd source, Mechanical Turk

## Abstract

Conversational User Interfaces (CUI) are widely used, with about 1.8 billion users worldwide in 2020. For designing and building CUI, dialogue designers have to decide on how the CUI communicates with users and what dialogue strategies to pursue (e.g. reactive vs. proactive). Dialogue strategies can be evaluated in user tests by comparing user perceptions and reactions to different dialogue strategies. Simulating CUI and running them online, for example on crowdsourcing websites, is an attractive avenue to collecting user perceptions and reactions, as they can be gathered time- and cost-effectively. However, developing and deploying a CUI on a crowd sourcing platform can be laborious and requires technical proficiency from researchers. We present Chatbot Language (CBL) as a framework to quickly develop and deploy CUI on crowd sourcing platforms, without requiring a technical background. CBL is a library with specialized CUI functionality, which is based on the high-level language JavaScript. In addition, CBL provides scripts that use the API of the crowd sourcing platform Mechanical Turk (MT) in order to (a) create MT Human Intelligence Tasks (HITs) and (b) retrieve the results of those HITs. We used CBL to run experiments on MT and present a sample workflow as well as an example experiment. CBL is freely available and we discuss how CBL can be used now and may be further developed in the future.

## Introduction

Users can interact with conversational user interfaces (CUI), using natural language, by posing questions like “Who was the first president of the United States?” or by giving commands like “Play some classical music please.” CUI then respond in turn using natural language, like “The first president of the United States was George Washington”, or “Okay, here is a playlist that you may like: Relaxing Classical Music.”

User interfaces that can be controlled conversationally, have become more popular over the past decade (Richter, 2016; Kinsella, 2019; Kuligowska, 2015; Mordor Intelligence, 2020). This is particularly pronounced in the rise of speech assistants (Richter, 2016; Kinsella, 2019) and chatbots (Kuligowska, 2015, 2020). In fact, commercial speech assistants were introduced in 2011 with Siri (Schonfeld, 2010) and in 2020 it is estimated that more than 1.6 billion people world-wide use speech assistants like Siri, Alexa, Google Assistant, Cortana and YuDian (Richter, 2016). Moreover, chatbots have been on the rise too (Mordor Intelligence, 2020). The conversational aspect is similar for chatbots and speech assistants. Companies use chatbots for example to support customers (Adamopoulou & Moussiades, 2020) and business interest is reflected in the expected growth of chatbots: In 2019 the chatbot market was valued at around 17 billion US dollars (USD) and the market is expected to reach a size of about USD 102 billion by 2025 (Mordor Intelligence, 2020). The broad adoption and growth of CUI suggests both business and societal interest in CUI. In addition, it emphasises the importance and timeliness of research into them. CUI involve human-computer interactions that are modelled after human-human conversations. There are many aspects of such conversational interactions with machines, that are of potential interest to social science research, including behavior, emotions, perceptions, linguistics, memory, usability, privacy and trust.

COVID-19 restricted personal contact throughout the world and affected the ability of social scientists to conduct their work by inviting participants to labs and interacting with them in person. Crowdsourcing platforms, like *Amazon Mechanical Turk* (*MT*), connect experimenters to participants remotely and allow them to gather behavioral data without inviting participants to the lab. Such platforms come with drawbacks and an in-detail discussion of the benefits and drawbacks of Mechanical Turk can be found in Chandler, Rosenzweig, Moss, Robinson, and Litman (2019) and in Buhrmester, Kwang, and Gosling (2016). Benefits of remote crowdsourcing include the ability to conduct experiments while observing social distancing, cost-efficiency and time-efficiency (Buhrmester et al., 2016).

MT is a platform for connecting requesters, who are customers of MT that create tasks for so-called workers, who are payed by requesters for completing their tasks. As a service to requesters, MT provides templates for quickly creating common tasks on MT, like writing a text-description of an image, or transcribing speech recordings. However, at the time of writing this article, there is no MT template for simulating CUI. Therefore, if researchers want to simulate CUI and deploy them on MT, they need to develop a custom solution. Developing such a bespoke solution requires technical proficiency that social science researchers may not have. With that in mind, we, a team consisting of a social and life scientist, an engineer and a software developer, have designed, developed and tested a framework for simulating chatbots and deploying them on MT, that can be used by researchers in the social sciences, like psychologists, sociologists and Human-Computer Interaction (HCI) professionals. We call this framework *Chatbot Language* (*CBL*) as it is a simple computer language based on JavaScript, that serves the specific purpose of simulating chatbots. CBL is client-based, that is it runs in the browser of users. CBL supports deployment of simulated chatbots on MT, but CBL chatbots can also be run offline, e.g. on a lab computer, or online on a private server. Thus CBL does not depend on MT, but MT is supported and can be used to quickly gather data.

In this article, we compare tools that are used by the dialogue system community with CBL to identify similarities and differences with CBL (see Section 2), then we give an overview on what researchers can do with CBL (see Section 3) and how they can set up experiments (see Section 4). Moreover, we present an example study with CBL (see Section 5). We end with a discussion on possibilities and limitations of CBL (see Section 6).

## How does CBL compare to other tools used by the dialogue system community?

Tools that are used by the dialogue system community can be compared along multiple dimensions. One dimension suggested by Papangelis et al., (2020) is whether their focus is on research, on production or both. Another dimension of system comparison are supported features. Finally, we find a distinction in terms of what the system is designed to achieve useful. Thus, in this section, we will compare a selected number of systems with CBL in terms of (1) focus on research and production, (2) features and (3) what the system is designed to achieve.

We compare the following tools:

- DialCrowd (Lee, Zhao, Black, & Eskenazi, 2018)
- ConvLab (Lee et al., 2019)
- ParlAI (Miller et al., 2017)
- Plato Dialogue System (Papangelis et al., 2020)
- Dialogflow (Cloud, 2017)
- Lex (Amazon AWS, 2017)
- AIML (Wallace, 2011)

This is not a comprehensive list of tools used by the dialogue system community and we chose these tools to lay out the landscape of tools used by the dialog system community and position CBL in that landscape. For this, we chose tools that are similar to CBL and, at the same time, may differ in type, in features (see Table 1) and intended purpose. Our selection of tools is not exhaustive and we did not conduct a systematic literature review of tools in the dialogue system community.

The tools that we chose for comparison are popular and some have been compared previously (Papangelis et al., 2020).

**Table 1:** This table shows selected features of tools used by the dialogue system community. In the table the abbreviation “DS” stands for dialogue system and the abbreviation “MT” stands for Mechanical Turk. A “✓” indicates that this feature is supported by a tool and a “✕” means that a feature is not supported by a tool

Tools	Build DS	Surveys	MT
DialCrowd	✕	✓	✓
ConvLab	✓	✕	✓
ParlAI	✓	✕	✓
Plato Dialogue System	✓	✕	✕
DialogFlow	✓	✕	✕
Lex	✓	✕	✕
AIML	✓	✕	✕
CBL	✓	✓	✓

DialCrowd is a toolkit for dialogue system assessment (Lee et al., 2018). ConvLab (Lee et al., 2019), ParlAI (Miller et al., 2017), Plato Dialogue System (Papangelis et al., 2020), Dialogflow (Cloud, 2017) and Lex (Amazon AWS, 2017) are platforms for dialog system research or production. A more detailed distinction of intended production and research purposes of these systems can be found in Papangelis et al., (2020).

AIML is a markup language and CBL is a programming language. AIML is a subset of the markup language XML (Wallace, 2011) and designed for describing chatbots. The capabilities of AIML are limited to what can be described using that XML subset. CBL is based on JavaScript and JavaScript is a turing-complete language whereas XML is not turing-complete. This means that CBL is more expressive and powerful as it has access to all JavaScript functionality and is not limited to an XML schema. In addition, JavaScript possesses a large library ecosystem and access to functionality from the browser (e.g. playing audio, text-to-speech, access to external APIs etc.). For example, JavaScript provides open-source Natural Language Processing (NLP) packages (Graype Systems 2017; AXA Group Operations Spain S.A. 2018; Kiro Risk 2014) that enable – among others – fuzzy matching, intent and slot recognition. CBL is not designed to be used with external NLP packages. However, it is possible to use NLP packages in CBL, for example by using the function s.match_if (for an example of usage see Table 4 in the Appendix). Note, that using NLP packages would require a level of expertise beyond that of the average intended user, which include social scientists.

Note that AIML interpreter exist in JavaScript (e.g. https://www.npmjs.com/package/aimlinterpreter) and they could be used to provide AIML with the ability to execute JavaScript code and access functionality from the browser. This means that chatbots designed with AIML could be used in the CBL framework. CBL is the only tool based on JavaScript from the tools that we compare.

Unlike the other tools, AIML and CBL do not provide Graphical User Interfaces (GUI). Importantly, neither AIML nor CBL are designed as platforms or toolkits with a GUI, but as scripting languages.

Section 3 gives a detailed overview of features supported by CBL. In contrast, Table 1 picks out three features to compare across tools used by the dialogues system community. We chose these three features to highlight differences between tools and introduce the concept of intended purpose. The three features we selected are: (1) allows users to build a dialogue system, (2) supports the creation of surveys and (3) supports crowd-sourcing via Mechanical Turk. CBL supports these three features as they help to meet it’s intended purpose: informing dialogue design decisions. CBL was designed to assess user behavior and perceptions in response to interactions with dialogue systems (Brüggemeier, & Lalone, 2022a). Specificially, CBL was designed to present users with dialogue systems that differ only in the design of the dialogue to assess if and how dialogue design might affect users. In a recent study that we conducted with CBL, we found that differences in dialogue design affect behavior and perceptions significantly. Therefore, we believe that CBL will be useful for the dialogue system community. Importantly, none of the other tools that have similarities with CBL, supports all of these three features. We argue that this is because they differ in their intended purpose.

One can roughly distinguish two purposes for tools used by the dialogue system community: production and research (see e.g. Papangelis et al., 2020). ParlAI, Plato, DialogFlow, Lex and AIML support building and deploying production systems, whereas DialCrowd, ConvLab and CBL do not. Notably, the tools that support production can be used for research also. Research can be further differentiated to include collection and validation of training data, testing of different system architectures and testing of dialogue design. This is not a comprehensive list of research focuses for dialogue systems, but we found these three categories useful to distinguish research purposes of the compared tools. Importantly, this is also not an overview of what can be achieved with tools. For example, tools that are not designed for collection of training data for Machine Learning (ML), can be used to collect such data nevertheless. However, dialogue data that are collected with tools not designed for data collection for ML, may provide data in a format that requires post-processing to make it usable in a ML pipeline. In contrast, tools, that are designed for collecting data for ML training, may already include the necessary post-processing steps and a connection to a ML pipeline. DialCrowd, ConvLab, ParlAI and Plato are designed for collecting training data for ML. DialogFlow, Lex, AIML and CBL are not designed for collecting training data for ML. However, it may be possible to use these tools for collecting training data for ML also, by adding post-processing and connection to a ML pipeline.

ConvLab, ParlAI and Plato are designed for testing effects of different system architectures. For example, different Natural Language Understanding (NLU) models may affect user perceptions and behavior. These tools are designed to allow researchers to easily implement and compare different system architectures. Other tools could be used for such research also. For example, CBL allows researchers to implement different NLU models that are supported in JavaScript. Effects of those NLU models could be compared using CBL. However, CBL is not optimized for this type of research and lacks support functions.

Notably, CBL is the only tool, except DialCrowd, that meets the intended purpose of dialogue design research. While both CBL and DialCrowd can be assigned the purpose of dialogue design research, they differ in the means they provide for such research: CBL is designed to evaluate user behavior and perceptions after interactions with dialogue systems, whereas DialCrowd is designed to evaluate system responses without interacting with dialogue systems (Lee et al., 2018). Note that DialCrowd supports connection to interactive dialogue systems, however they can not be build within DialCrowd. Instead, dialogue systems have to be build with other tools and deployed on servers so they can be accessed within experiments build with DialCrowd. This design has implications for crowd sourcing data on interactions with dialogue systems with DialCrowd. Workers have to follow a link to access the dialogue system that is to be evaluated and, after finishing their interaction, have to go back to the crowd-sourcing platform and verify, e.g. with a code, that they interacted with the dialogue system, so that they can get paid. This can create at least two issues: (1) workers may fail to transition successfully between the crowd-sourcing platform and the dialogue system and (2) researchers may fail to correctly assign data from the crowd-sourcing platform to interactions with the dialogue system. CBL is designed to avoid these issues. CBL lets users build an HTML page with an interactive dialogue system. CBL then uses an *iframe* to embed the HTML page with the dialogue system into the HTML page of Mechanical Turk. This means that workers see and interact with the CBL dialogue system within Mechanical Turk. They stay on the Mechanical Turk webpage throughout the experiment and all data, including interaction data and data from Mechanical Turk like HIT ID and worker ID, are gathered in one place.

Notably, other tools could be used for dialogue design research also. For example in Plato, one could tweak reward functions for reinforcement learning which will lead to variations of the same system. For example, one version could be trained to avoid confirmations by implementing a negative reward when the system confirms. Another version could be designed to request and confirm information before moving to the next piece of information by implementing a penalty for attempting to ask for something new without confirmation. Thus, one could test whether users prefer a system that could be annoying because it asks for confirmation in every interaction versus a system that may be less annoying but can lead to an incorrect and potentially detrimental result by collecting the erroneous information. The same is true for other tools. Importantly, external surveys would have to be used to assess user perceptions.

## What can I do with CBL?

CBL is an open source framework for simulating chatbots. We intend CBL to be used for research and development of CUI. For example, CBL makes it possible to source sample dialogues for designing or training a system. Moreover, CBL can be used to create multiple experimental conditions that are randomly sampled and then presented to MT workers, which allows controlled experimental design. In addition, CBL can be utilized to create instruction pages that are presented to MT workers before interacting with chatbots. Such instructions can inform MT workers about data protection and their rights as participants, thereby abiding to data protection regulations and to ethical standards of research (European Data Protection Supervisor, 2020). In addition, instructions can inform participants about the task that they are to complete. Note, that both instructions and chatbot interactions can be experimentally exploited, by randomly assigning different conditions to workers. Furthermore, CBL can create surveys that are presented after chatbot interactions and can collect data on user perceptions and reactions to chatbot interactions and instructions. By open-sourcing CBL, we make it available for the developer community for contributions and modifications (https://github.com/audiolabs/cbl). CBL is published under a modified BSD license and is compatible with commercial as well as non-commercial usage. CBL makes use of third-party open-source software (OSS) components. The OSS components are libraries that are installed when the user follows the installation instructions and they interact directly with CBL. Some scripts included with CBL communicate with external services such as Amazon Web Services (AWS). This is relevant when intending to use CBL – especially for commercial purposes – the third-party OSS licenses should be checked, as they might affect usage obligations. We provide the used third-party licenses in our repository (Lalone & Brüggemeier, 2021).

In it’s current state, CBL can be used in a lot of different applications and we outline here parameters that can be modified with CBL.

### Choose language

CBL can be used to simulate chatbots in any language that can be encoded with UTF-8, which essentially means any naturally spoken language. We have tested CBL in English and in German.

### Show introduction text

CBL users can create custom introduction texts, to inform participants about their rights and their tasks. Importantly, CBL can present multiple introduction sites consecutively. For example, experimenters may choose to show data protection information on a first introduction screen, followed by a second introduction site with instructions about the task. When participants accept the data protection agreement, they can click a continue button and then see the task instructions. Moreover, experimenters can choose to present different instructions to different participant groups, thereby testing effects of instructions on interactions and perceptions of chatbots.

### Implement chatbots

With CBL, experimenters can control what a chatbot says and what key words the bot reacts to, thereby controlling the dialogue flow. Notably, MT workers can respond freely to every chatbot utterance, thus providing varied dialogue data.

CBL uses regular expressions to detect keywords in worker input. Consider for example, a banking scenario in which workers are asked to use a chatbot to check credit balance. In this use case, experimenters can define expected keywords like ‘check’, ‘balance’ and ‘credit’ (Brüggemeier, & Lalone, 2022a). If one of these keywords is detected, the dialogue progresses and a chatbot response is triggered.

However, not every user input can be expected, therefore we included the CBL command unknown that enables experimenters to deal with unknown user input (for more information see Appendix A.2.2). With this CBL command experimenters can define how a chatbot should react to an unexpected participant query or command. For example, we may expect participants to check credit balance, however instead they may attempt to do small talk with the chatbot and ask “Hey, how are you today?”. In that case, the unknown command in CBL can be used to define a response like “Sorry, I can’t help you with that. Try checking your credit balance.” Notably, this approach allows unexpected input and at the same time guides participants to successfully completing an interaction. Participants can be guided with instructions, with a task prompt and with the chatbot dialogue, which can all be defined by experimenters.

### Choose output modality

CBL includes an optional *text-to-speech* (*TTS*) feature, that supports reading out-loud of text with an artificially generated voice that is presented to MT workers. This text can be instruction text, or chatbot utterances. Currently, CBL supports text-only presentation or combined text and audio presentation of chatbot utterances and instructions. CBL users may choose to give their chatbots a voice and maintain instructions in text-only mode, and vice versa. By adding a voice to a chatbot, researchers can investigate effects voice may have on user experience and user trust (Burri, 2018).

### Choose voice

CBL supports two types of voice generation: (1) pre-generating voices, that is generating voice audio files before running the chatbot interaction on MT and (2) real-time voice generation, that is creating voice output at the time when a worker interacts with the CBL experiment. In this second case, voices are generated on the computer of the worker by the browser the worker uses. Importantly, browser-based voices depend on the browser and on browser settings and therefore can vary from worker to worker. Thus, if an experimenter wishes to control what voice workers will hear, they may want to opt for pre-generated voices.

For pre-generating voices, CBL connects with one of two TTS services: Google translate TTS and Amazon Polly. Note that these services may charge for voice generation and may require the creation of an account. In addition, other TTS services can be used to create voices and use them with CBL. Thus, CBL does not require its users to utilize the supported TTS services. Note, that the supported services provide voices in a number of languages and dialects. For example Amazon Polly supports 29 languages and dialects (Amazon AWS 2020).

### Playback audio

In addition to supporting reading out loud of text with artificial voices, CBL also supports playback of any mp3 file, including music. For playing back audio files that are not artificial voices, experimenters can use the command play_audio (see Appendix A.2.2). Conversely, ‘stop_audio’ stops playback. This playback function can be useful for use cases in which participants are supposed to control media (e.g. music, radio, podcasts) with a chatbot.

### Implement survey

Experimenters can create surveys with CBL that are integrated in the experimental flow. After completing an interaction with a chatbot, workers can be automatically forwarded to a survey page.

CBL currently supports four types of survey items: (1) text-entry boxes, (2) drop-down lists, (3) semantic differential scales and (4) Likert scales. For more details see Appendix A.2.2. Experimenters can choose a custom number of points for both Likert and semantic differential scales. Moreover, workers are required to select points for each scale item to complete the survey. In contrast, by default text-entry is not required to complete a survey in CBL. Other types of survey items and survey design can be implemented with CBL, however this requires some programming knowledge.

### Randomize presentation of experimental conditions

For randomized controlled trials, experimenters need to assign participants randomly to control and experimental conditions. This can be achieved manually, e.g. in lab settings, by alternating conditions participants are presented with. When running experiments on the third-party service MT, the MT requester User Interface (UI) allows requesters to assign workers automatically to conditions. However, this is only true for experiments that can be set up with the MT requester UI. Currently, chatbot interactions can not be set up with the MT requester UI. CBL bypasses this, by constituting a framework to set up chatbots and leveraging MT API to run these chatbots on the crowdsourcing platform. CBL supports random assignment to experimental conditions. Note, that CBL can be used not only for randomized controlled trials, but also for observational studies and for gathering training data.

### Run online or offline

CBL experiments can run online and offline. In it’s current state, CBL supports online data collection via Mechanical Turk APIs. It is possible to extend CBL to support other crowdsourcing platforms like Prolific (2021). Currently, APIs of these sites are not supported in CBL. Moreover, CBL experiments can be run on a private server and distributed with links. Thus, running CBL online does not require using a third-party service.

In addition, CBL experiments can be implemented on a local computer and used for local data collection. This means, that it is not necessary to set up CBL experiments online, but a local computer is sufficient. This may be useful for studies that are planned in a lab setting. CBL files required for running the experiment (compiled HTML-file and, where applicable, audio files) need to be stored on a computer that can be accessed by participants (e.g. a laboratory laptop). In this local mode, the results of the experiment can be printed at the end of the experiment for the experimenter to copy and save. Moreover, a compiled HTML version of a CBL experiment can be sent to participants to run on their personal computers. In that case, participants need to be instructed to send back the results to the experimenter. If a compiled HTML version is sent to participants, experimenters need to make sure that audio files used by CBL are publicly available. Running CBL experiments locally may be attractive for enhancing privacy of participants, as data may not be stored on third-party servers.

## How can I set up CBL experiments?

This section includes an exemplary walk-through for running an experiment with CBL in Section 4.1. Moreover, we present takeaways from interviews with practitioners of CBL in Section 4.2. A detailed overview of CBL syntax and semantics can be found in Appendix A

After reading this section, it should be possible to evaluate the use of CBL for a research project, plan a CBL experiment and to implement and run it.

### Example walk-through

Here we showcase a typical workflow of running experiments with CBL. An example of results gathered with CBL can be found in Section 5 and in Brüggemeier, & Lalone, (2022a). While the results in Section 5 and in Brüggemeier, & Lalone, (2022a) give practical insight in how CBL can be used for research, we deem it useful to present a generic workflow of experimentation with CBL: A description of a workflow from design to technical implementation to data analysis is not typically part of research papers, however it is useful to practitioners who want to use CBL as a research method.

**Fig. 1:**
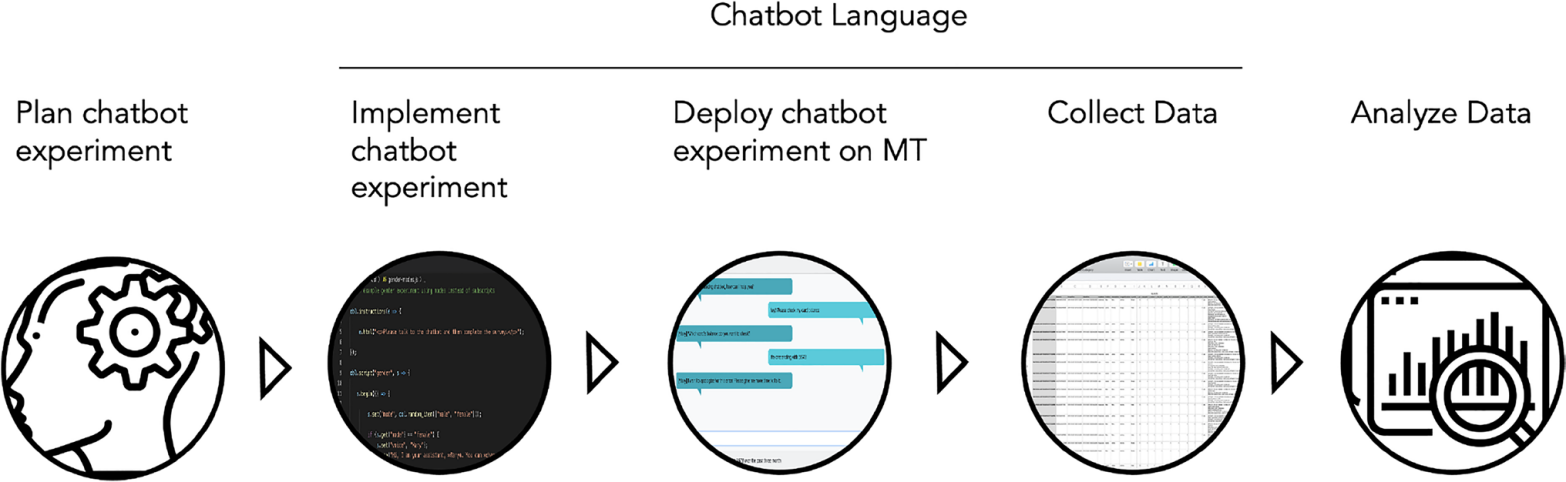
Simplified illustration of a workflow involving Chatbot Language (CBL)

A typical workflow of an experimenter using CBL includes (see Fig. 1):

1. planning the chatbot experiment,
2. implementing the chatbot experiment with CBL,
3. deploying the CBL file on MT,
4. collecting data from MT using CBL,
5. analysing the data.

Notably, only points 2 to 4 are completed with CBL. However, as planning experiments with CBL (point 1) benefits from insights into CBL, we will briefly discuss this point also.

#### Planning

When designing an experiment with CBL, researchers should consider the three distinct parts of a CBL experiment: instructions, chatbot interaction and survey. Each of these parts is optional. Note, however, that the order of these three parts can not be changed.

If experimenters plan to run a study with multiple conditions, they may want to make use of the CBL set and random_item command, that enable users to set conditions that are then randomly selected in the course of each chatbot interaction.

Importantly, when designing chatbot interactions, experimenters should follow best practices of conversational design that are for example discussed in Pearl (2016) and Moore, Arar, Ren, and Szymanski (2017). Similarly, when designing the post-interaction survey, designers may wish to follow survey design principles as outlined in Blair, Czaja, and Blair 2013.

When designing experiments with CBL, experimenters may want to consult Section 3 in this article, as it describes which experimental elements can be modified using CBL.

#### Implementing

As CBL is a high-level programming language, implementing experiments with CBL requires usage of a code or text editor, such as Visual Studio Code, Atom or Vim. Our CBL GitHub repository (Lalone & Brüggemeier, 2021) includes sample code that showcases how to include instructions, define dialogue flows, set voices and design surveys with CBL.

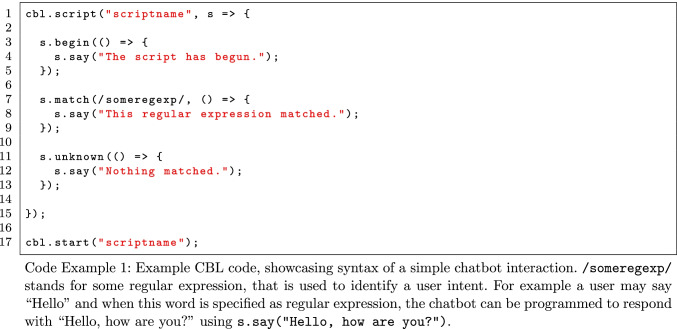



If experimenters pre-generate voice files or want to play back other mp3 files (see Section 3.6), these sound files need to be made accessible online. This is because CBL experiments are client-based, that is they run in the browser of the client, in this case MT workers. In order for the MT workers to be able to hear sound files, their browser needs to be able to access the files via the Internet. To enable online access of sound files, experimenters can for example create an Amazon Web Services (AWS) server instance and upload the sound files there, then copy the link to the server instance into their CBL script, thus indicating where to find the sound files. A detailed step-by-step instruction for this process is given in the Readme of our GitHub repository (Lalone & Brüggemeier, 2021).

Code Example 1 shows a sample implementation of a simple chatbot interaction. For a more detailed overview of syntax and semantics of CBL see Appendix A. In Appendix A you also find Code Example 2 which shows a simple implementation of a questionnaire.

#### Deploying

Once an experiment is implemented in CBL, it can be compiled into an HTML page. Detailed instructions for building and deploying a CBL script can be found in our repository (Lalone & Brüggemeier, 2021).

The HTML file can be opened and tested locally, which enables experimenters to evaluate their experiment before publishing it on MT. After testing the experiment locally, the HTML file can be uploaded either directly to MT, or to the MT Sandbox environment. MT Sandbox lets requesters run and test their applications on MT servers, prior to publishing them for MT workers (Amazon Mechanical Turk, 2020). Testing CBL experiments in the MT Sandbox environment is optional, as is testing them locally.

Eventually, when experimenters are satisfied with their CBL experiment, they can deploy it on MT and thus publish it to the MT worker community. Experimenters need to define the number of workers that can take part in the experiment, the compensation MT workers receive for participating in their experiment, as well as keywords that may help workers to find the experiment on the MT platform. In addition, experimenters need to specify worker inclusion or exclusion criteria. For example, we excluded workers who took part in prior chatbot experiments. One can also include workers who are based in a specific country only, for example the United States. More information on how certain worker groups can be excluded or included and how to specify this with CBL, can be found in our Readme (Lalone & Brüggemeier, 2021).

**Fig. 2:**
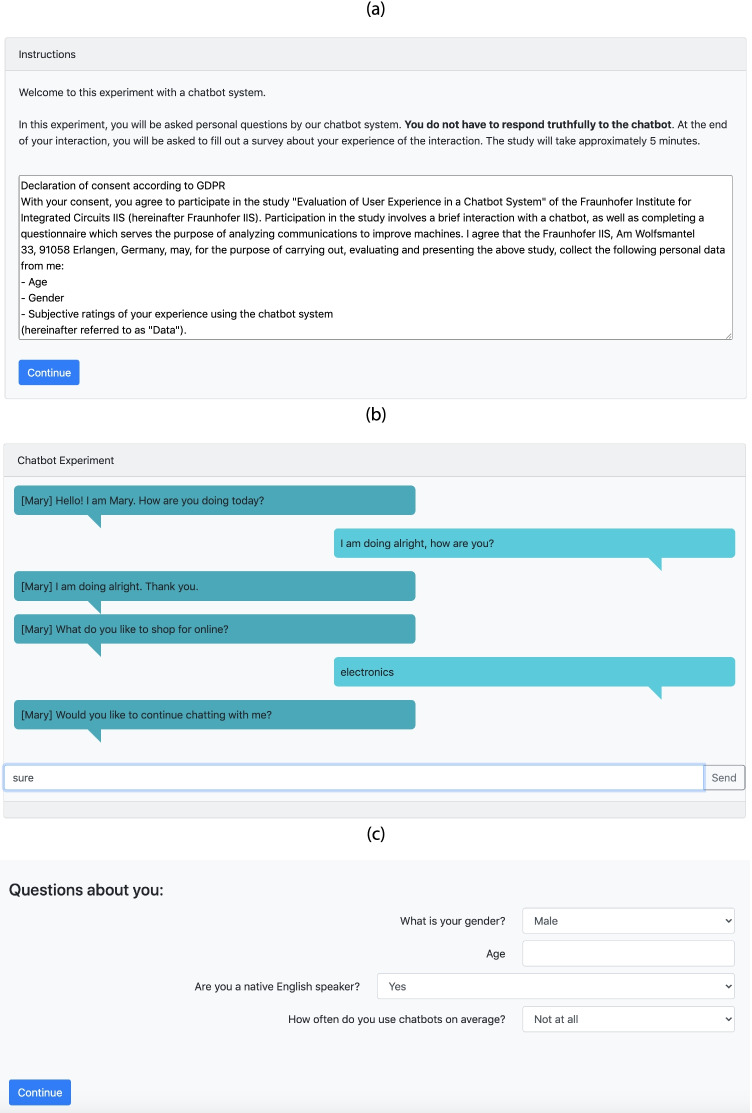
Screenshots of a sample implementation in HTML of a CBL experiment, showing (**a**) an introduction screen, (**b**) an ongoing interaction with a chatbot and (**c**) survey questions

Figure 2 shows a sample introduction, a chatbot interaction and a survey created with CBL.

#### Collecting data

When a CBL experiment is published on MT it is assigned a HIT ID. This HIT ID is a unique identifier that can be used by CBL to pull results from the experiment, even while it is still running. To pull data with CBL, experimenters are required to use a command line interface and instructions are provided in our GitHub repository (Lalone & Brüggemeier, 2021).

CBL saves results as CSV file. Data include the HIT ID, the worker ID, which is a unique identifier for MT workers, and an assignment ID which is a unique identifier that links workers to HITs. This information can be useful for accepting or rejecting HITs or giving rewards to specific workers. Resources on how to do this are linked in our Readme (Lalone & Brüggemeier, 2021).

Moreover, data include timestamps of when a worker accepted the HIT and when they submitted the completed HIT. This information can be used to compute the average duration until completion of the experiment. Other relevant data are the transcript of the interaction, which is saved as string with labels indicating whether the user, i.e. the worker, or the chatbot are speaking, and survey results. For a detailed description and presentation of data collected with CBL see Brüggemeier, & Lalone, (2022a).

### Takeaways from test runs

CBL was tested by two psychologists and one HCI researcher with no prior knowledge in JavaScript and no formal training in programming. In addition, CBL was tested by one computer scientist and two engineers with minimal prior experience in JavaScript. All testers were between 20 and 35 years of age. Testers were able to create chatbots with CBL within one afternoon up to a week of casual attempts using CBL.

Thus, we conclude that CBL can be used by researchers with no prior knowledge of JavaScript and little background in programming.

We asked three testers about what they used CBL for and what CBL’s purpose was in their opinions. CBL was described as a tool to evaluate dialogue strategies with behavioral and attitudinal measures. Interviewee I3 gives a concise summary of CBL’s purpose from their point of view: *“I would use CBL for studies where you want to look at dialogues and compare dialogue strategies. It is a testing tool.” *We ran the experiments, that we created with CBL, with more than 3500 MT workers, who successfully completed them. This suggests that experiments created with CBL are sufficiently usable that MT workers can complete them. In addition, it is notable that 100 to 400 MT workers completed online experiments within six hours of putting an experiment online on MT, which speaks to the time-efficiency of collecting data with CBL and MT. Interviewees also highlighted efficiency of CBL. Note, we did not restrict worker participation based on their region, but excluded workers that previously took part only. If experimenters require participants to be based in certain regions this would reduce the number of workers that can take part and thus may affect the time-efficiency of data collection.

Practitioners also remarked on the process of building and testing experiments with CBL. Interviewee I2 mentioned that they found working with CBL intuitive. As CBL is JavaScript based, JavaScript can be used within CBL to customize experiments, as described by I3 *“I found it comforting that I could go into the framework and implement my own stuff, like that we capture time stamps, or that I adjust the button size.”*. Moreover, CBL experiments are compiled as HTML files and run in the browser. Interviewee I1 noted: *“Being able to run experiments on a web browser in different environments helped me to identify potential errors, like audio not being heard by people who switch off their audio. Then I was able to control for it by introducing a Yes/No question to the survey saying ‘I was able to hear the audio in the interaction’.”* Interviewees I3 and I1 highlighted ease of iteration with CBL. I1 noted: *“It is very very easy for iterating, for updating. You can correct errors in your experiment right away.”*

What is more, I1 highlighted that CBL provides all parts of experiments, including data protection statement, introduction, dialogue system interaction and survey in one place.

Practitioners also commented on where they saw potential for improvement for CBL, including more extensive documentation, which we added. Moreover, I3 noted that JavaScript can be used to expand functionalities of CBL, however this requires a level of expertise that extends that of the intended user. CBL has potential to be expanded by adding functions that make customisation easy for people without programming background. We open source CBL and hope that the dialogue system community will add functions that cater to their needs.

## Example study with CBL

We present an example study to illustrate how CBL can be used to inform dialogue design decisions empirically, based on user behaviour and perceptions. Our study aims to identify dialogue strategies that allow designers to ask for personal data in a dialogue while respecting users’ privacy and maintaining system usability.

Users are regularly asked to disclose personal information to devices. Some CUI let users enter their information and other CUI ask for access to users’ devices and data. In both cases, forcing the user to disclose can frustrate users and may lead to rejection of the service (Leschanowsky, Brüggemeier, & Peters, 2021). In our experiment we aim to assess user behaviour and perceptions when a chatbot asks to access data vs when a chatbot asks users to enter data. We are especially interested whether the two strategies differ in users’ perception of privacy and their reported levels of frustration. In order to be able to generalize across contexts we investigate both strategies (enter and access of data) in two chatbot scenarios, a banking chatbot asking for users’ credit card number and a chatbot asking for location information (see Appendix B.1 for the dialogue trees of both chatbots). The two scenarios were chosen with distinct levels of information sensitivity in mind (Schomakers, Lidynia, Müllmann, & Ziefle, 2019).

In both the access and the enter condition, we give users the option to refuse sharing their data, for example by saying “no” to the question of whether data can be accessed or when asking them to enter personal data. Additionally, participants can protect themselves in the enter condition by providing misinformation. To be able to assess the number of participants who stated incorrect information, we asked them at the beginning of the survey whether they shared true information or misinformation.

We used objective and subjective measures to assess peoples’ perception and behaviour. Subjective measures included privacy perceptions and usability that were used before in experiments with CBL on conversational privacy (Brüggemeier, & Lalone, 2022b). Moreover, we assessed frustration. Our dialogues are designed such that no service is provided due to technical difficulties or closure of the restaurant (see Appendix B.1). This is based on the assumption that a positive ending (e.g. providing a fake balance in the banking scenario or telling the user that the pizza is on its way) might confuse or even upset users. The users might be unsure whether the fake credit card balance is actually correct or whether a pizza will be delivered to their location. To avoid this, we end the interaction by saying that no services will be provided. However, failure to provide a service could leave users with a feeling of frustration (Smith & Ellsworth, 1985). Thus we measure user frustration.

In addition, we are interested in timing of user decisions. Taking time to make a decision can lead to better and more informed decision making (Kahneman, 2011). Thus, we adapted CBL to capture users’ reaction and completion times during the interaction with the chatbot.

Moreover, we included three screening questions in our survey to check the reliability of submitted responses and whether participants paid attention to the survey questions. The survey of the study including the individual items and scales can be found in Appendix B.2.

**Table 2:** Summary of demographic and experimental data for the banking and location scenario

Demographic and experimental data	Banking	Location
# conditions	2	2
# participants	100	100
# excluded participants	7	16
# accepted participants in the access condition	50	41
# accepted participants in the enter condition	43	43
Mean (SD) age of workers in years	32 (9)	33 (9)
# Gender (female/male/diverse/not provided)	62/31/0/0	37/47/0/0
# Native English speakers (yes/no)	92/1	83/1
# Usage (weekly/monthly/less than once a month/never)	6/41/25/21	16/31/22/15

We set up our experiment in CBL following the workflow suggested in Fig. 1 and deployed the experiment on Mechanical Turk. We show dialogue flows for both scenarios and both conditions in Appendix B.1. Before the interaction with the chatbot, workers saw an instruction page that informed them about their rights as participants and introduced them to the task. After the interaction with the dialogue system, workers completed our survey (see Appendix B.2). We give an overview of demographic and experimental data in Table 2. Workers were paid $2 for their participation and took an average of 12 minutes to complete the experiment. This translates to an average hourly pay of $10. Participants who failed one or more screening questions were excluded from the analysis.

**Fig. 3:**
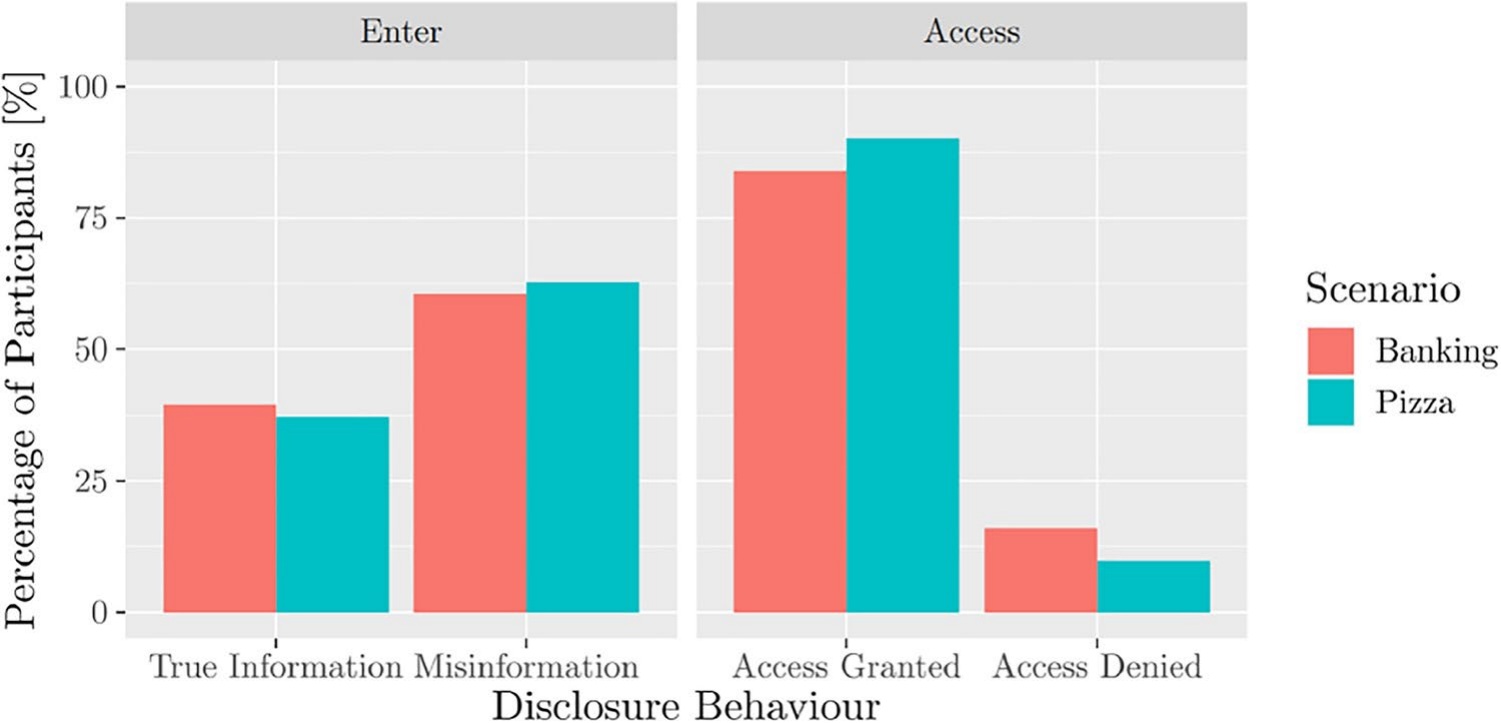
Percentage of participants providing misinformation and true information in the enter condition and granting or denying access in the access condition, displayed for both scenarios

In Figure 3 one can see that most of the participants exposed to the access condition gave access to their data while only roughly 40% shared true information in the enter condition. Interestingly, this is true for both scenarios. Further, we analyzed the transcripts from the enter condition manually and found that none of the participants in the location scenario explicitly stated “no” to providing information. However, more than 25% of participants exposed to the enter condition in the banking scenario specifically expressed that they did not want to enter personal information. Sometimes they even stated that credit card information is personal information as a reason for non-disclosure.

**Fig. 4:**
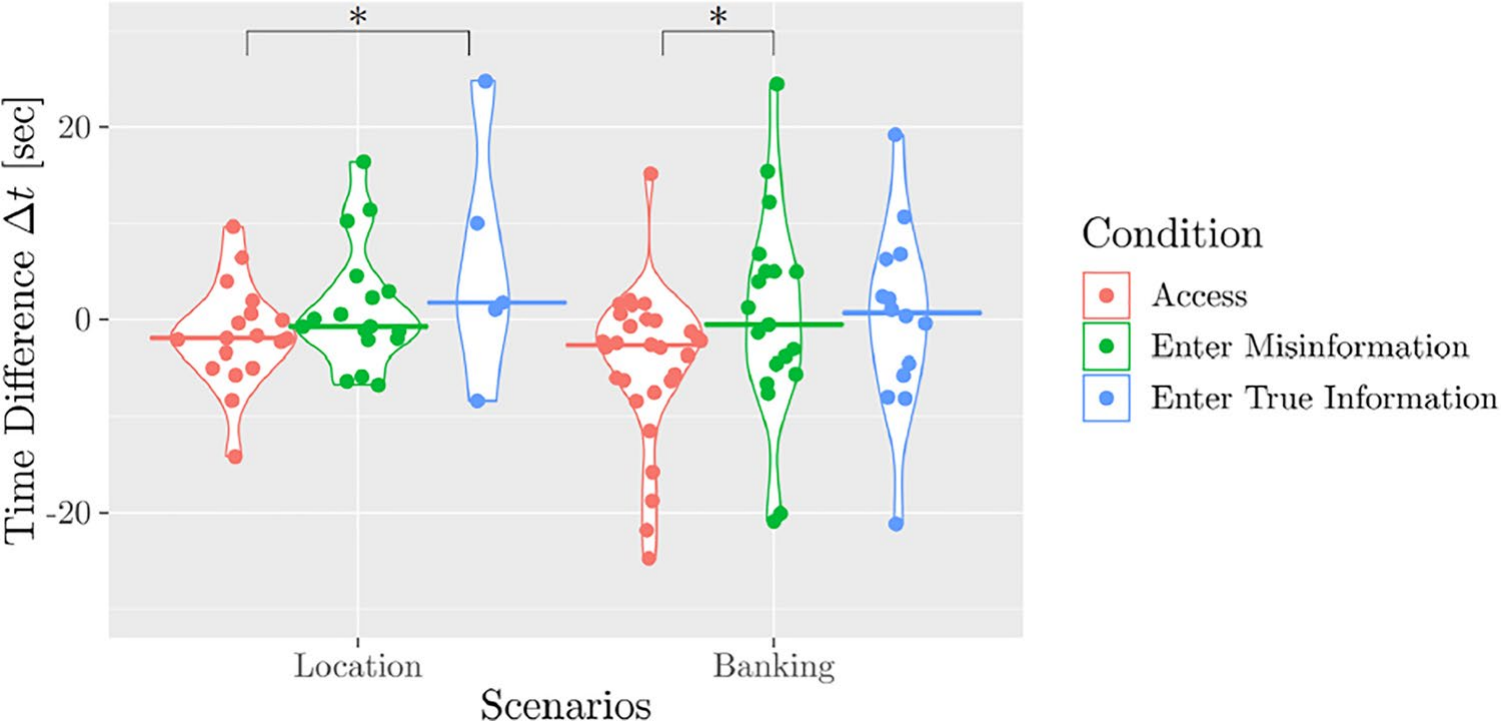
Times users take to respond to chatbot prompts in access and enter conditions. Times are displayed relative to the baseline response to prior chatbot prompts. 17 outliers are not visible in this figure in order to allow a close-up comparison of group differences. Median values are indicated by colored lines. Condition differences highlighted with an asterisk have a p-value lower than 0.025 in the pairwise comparison of conditions in each scenario as we apply Bonferroni correction to account for multiple testing

We find that the odds of disclosure are 3.35 times greater for the access condition than for the enter condition. Moreover, the odds of participants being frustrated in the access condition were 3.45 higher than in the enter condition. No significant effect was found for measures on privacy perception and usability.

We captured timestamps whenever the chatbot sent responses and when participants started typing. We compared the time participants took to answer to the question to disclose information and the average time they needed to reply to the remaining questions asked by the chatbot. The time difference ${\Delta } t$, the difference between this baseline and the time taken to respond to the condition-specific question, was computed for the sets available and results are shown in Fig. 4. Positive values indicate that participants took more time to respond to the chatbot’s prompt to enter or access data than to prior prompts, while negative values indicate that participants took less time to respond to data access or enter prompts than to prior prompts (see Appendix B.1). We find that in the access condition participants took less time before typing their response than in the enter condition.

We find that when participants are asked to enter personal data, they take more time before responding, they are more likely to choose not to share their data and they are less frustrated after an interaction with a dialogue system that asked for data. Moreover, we find that asking people to enter their data does not affect the perceived usability of a dialogue system. Hence, we conclude that dialogues that require personal information can be designed to be both usable and privacy-friendly by asking users to enter their personal information rather than asking for data access.

## Discussion

Chatbot Language (CBL) is an open-sourced framework under modified BSD licence for running interactive chatbot experiments. CBL supports deployment on Mechanical Turk. This is particularly useful (1) when wanting to obtain data remotely, (2) when choosing to diversify the participant base for experiments (Buhrmester et al., 2016) and (3) when needing to gather data quickly. For example, in a recent study (Brüggemeier, & Lalone, 2022a), conducted with CBL, we were able to gather data of about 400 participants in less than a day.

CBL can be used for a range of research projects, including

- designing dialogues of conversational user interfaces, for example by testing one dialogue design versus another in A/B testing,
- comparing effects of input modalities, e.g. by running an experiment with a text-based chatbot and comparing user perceptions or behavior to running the same experiment with a speaking chatbot,
- investigating effects of errors on user experience (Yuan, Brüggemeier, Hillmann, & Michael, 2020),
- examining effects of privacy violations (Sannon, Stoll, DiFranzo, Jung, & Bazarova, 2020) or conversational privacy (Brüggemeier, & Lalone, 2022a),
- studying effects of different voices on user behaviour (like male, female and neutral voices (Lee, Nass, & Brave, 2000); or voices with different accents, e.g. British vs. American accents (Tamagawa, Watson, & Kuo, 2011)),
- and investigating effects of different languages and cultures (Cambre, & Kulkarni, 2019).

This is not a comprehensive list of research projects that can be approached with CBL, but is intended to showcase application examples. However, we believe that the research community will come up with more applications than what we consider in this listing.

An open research question that is of interest both to researchers and practitioners is: Are results gathered with CBL comparable with real-life chatbot interactions? The answer to this question will likely depend on the real-life chatbot application that CBL chatbots are compared with. For example, a chatbot application that is running on a website that users can access from home, may be more comparable to CBL chatbots that are accessed via a website from home, than a chatbot application, that is running on a UI of an ATM, that can be accessed only within a bank. Use context likely matters. Moreover, there are roughly two types of chatbot systems: rule-based and machine-learning-based systems. CBL is a rule-based system and thus may render more similar results to other chatbots that are also rule-based than to machine-learning-based systems.

CBL chatbots are designed as simulations and embedded in an experimental framework that designates them as experimental, namely an instruction page and questionnaires. User behavior may differ when users know that they interact with a system in an experimental context rather than in a real-life context. Thus, we expect that CBL chatbots may render different results to real-life chatbots, even when use context and system type are matched. This means, that CBL chatbots can not replace experiments with real-life systems, however they may be an avenue to conduct conversational interaction experiments for researchers that do not have access to real-life chatbot data or do not have the resources to build real-life applications themselves. It remains to be investigated how comparable results from CBL interactions are to different real-life applications.

CBL supports real-time generation of audio. That means that it is not necessary to generate audio files in advance of running the experiment, but instead have them generated by the browser of the participants taking part in the study. However, this real-time generation of audio bears the caveat of providing no experimental control of the generated voice. For example, an experimenter may wish to conduct an experiment in English. When they run their experiment with real-time generation of audio, some of the participants may have browser settings that produce voices with a German Text-to-Speech (TTS) engine, thereby generating English sentences that might sound like a speaker with a heavy German accent. Importantly, having a German TTS generate audio of English text files may not sound like a speaker with an accent, but might be unintelligibly. This is true for other language combinations also. Therefore, we advise experimenters who want to control voice output to use audio files that were generated before running the experiment. CBL does support this option also (see Section 3.5 and Appendix A.2).

Moreover, other extensions to CBL are conceivable. For example, CBL currently detects user intents with regular expressions. Regular expressions are commonly used in Natural Language Processing (NLP), however they do not take into account the grammar or context of speech, which restrict their performance (Nadkarni, Ohno-Machado, & Chapman, 2011). Instead of using regular expressions for detecting user intents, it has become common in NLP to use artificial neural networks. Such Natural Language Understanding (NLU) models could interface with CBL to test them with users remotely. Moreover, it might be possible to extend CBL with support for Automatic Speech Recognition (ASR). ASR would allow users to communicate with CBL by using their voice instead of having to type text. Such interaction would be closer to interactions with speech assistants such as Amazon Alexa, Google Assistant and Apple’s Siri. Speech data that would be collected with CBL could be used to improve ASR models. However, both the integration of NLU and ASR models might impede real-time processing with CBL. CBL is client-based and runs in the browsers of participants. Custom NLU and ASR models might take too much processing to run in real-time on browsers. Thus, such models would either have to be optimized for running on browsers, or CBL would have to be extended to offer cloud support. Offering cloud support would be a major change to the current software layout of CBL. However, we would like to highlight that CBL can be used with existing JavaScript Natural Language Processing (NLP) packages (Graype Systems 2017; AXA Group Operations Spain S.A. 2018; Kiro Risk 2014).

## Conclusion

We present Chatbot Language (CBL), a high-level programming language based on JavaScript that enables researchers to crowdsource perceptions and reactions to dialogue systems in order to inform dialogue design decisions. Unlike other tools used in the dialogue system community, CBL is designed to inform dialogue design decisions and supports this with three main features: (1) building dialogue systems, (2) creating surveys and (3) supporting crowdsourcing via Mechanical Turk. We provide an overview of what can be done with CBL, explain semantics and syntax of CBL, demonstrate a sample walkthrough of running experiments with CBL, cite feedback from users of CBL and show results from an example study.

## Figures and Tables

**Fig. 5:**
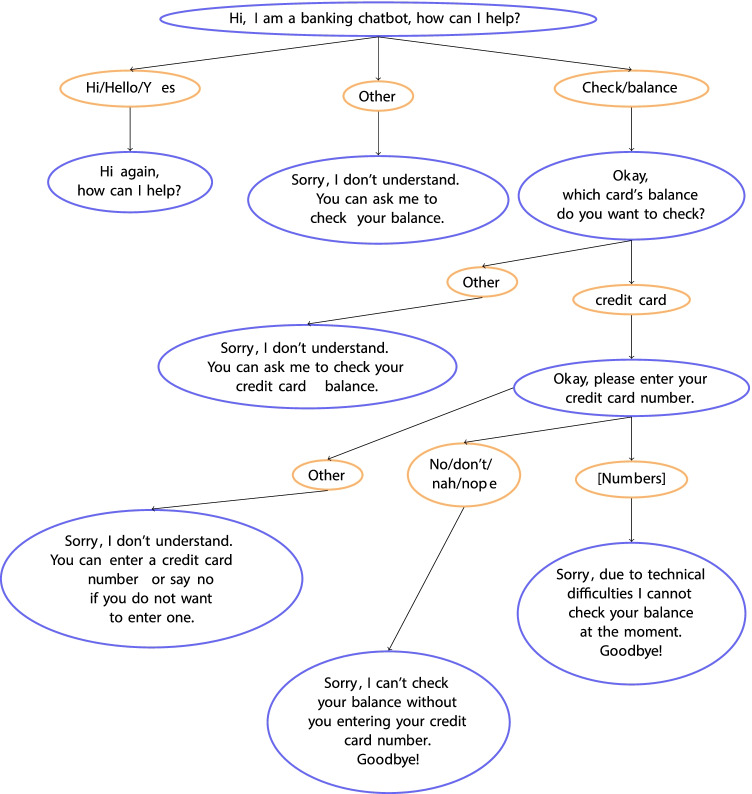
Dialogue Tree for the banking scenario in the enter condition, blue circle show the chatbot, orange circles show the possible inputs for the user

**Fig. 6:**
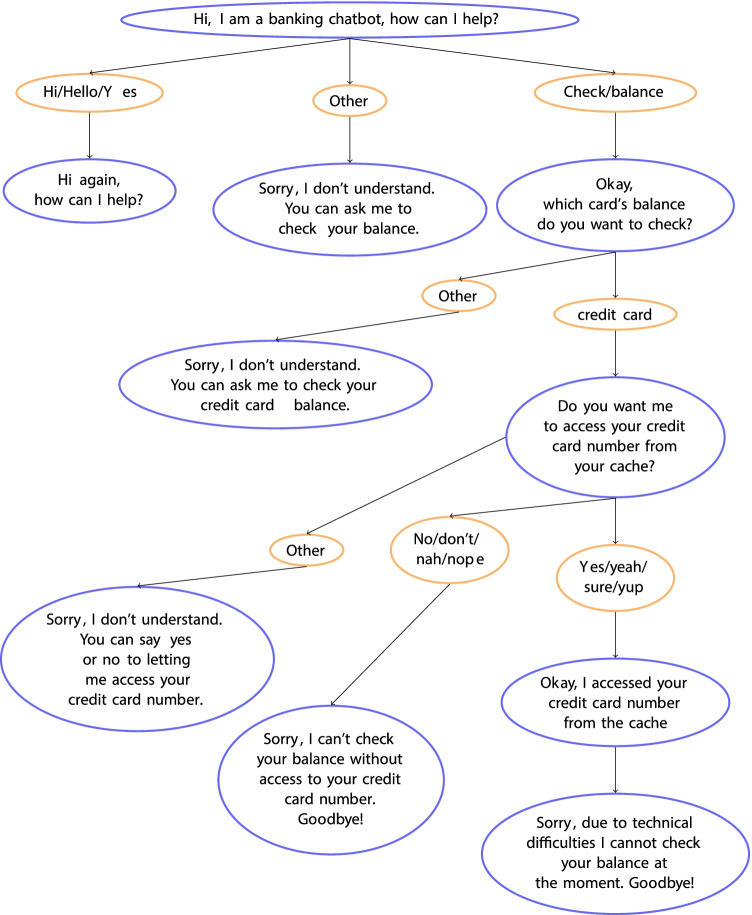
Dialogue Tree for the banking scenario in the access condition, blue circle show the chatbot, orange circles show the possible inputs for the user

**Fig. 7:**
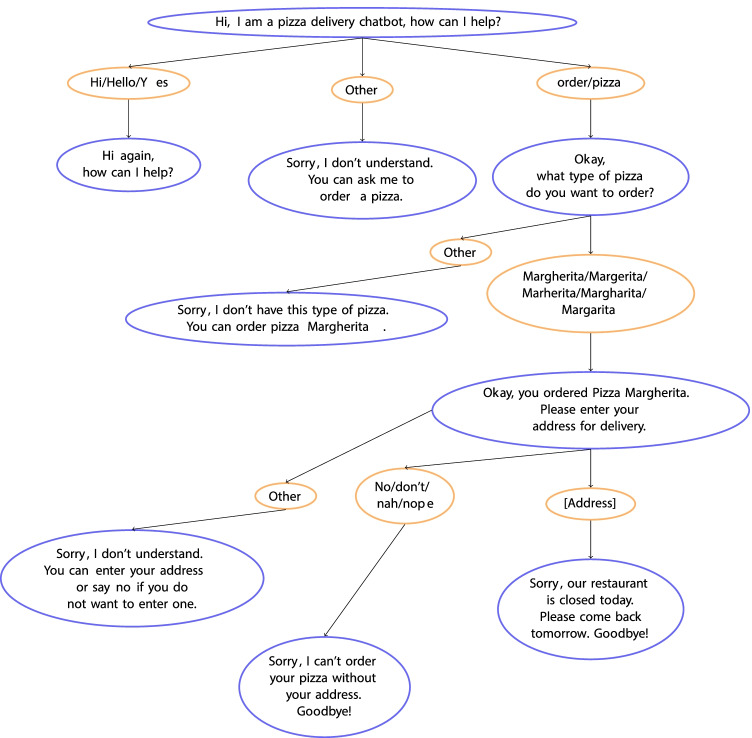
Dialogue Tree for the location scenario in the enter condition, blue circle show the chatbot, orange circles show the possible inputs for the user

**Fig. 8:**
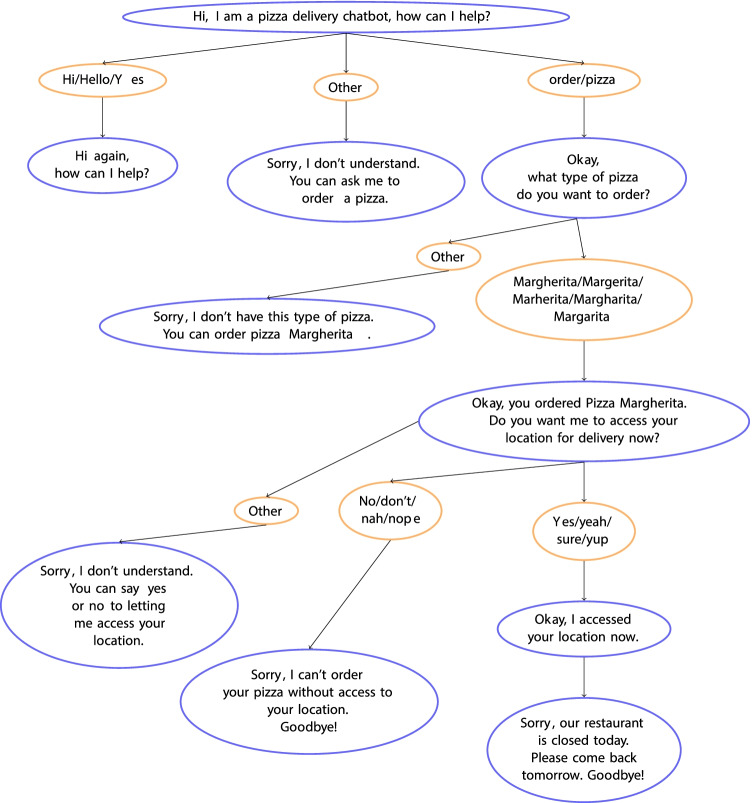
Dialogue Tree for the location scenario in the access condition, blue circle show the chatbot, orange circles show the possible inputs for the user

## Appendix A: Syntax and semantics

CBL is an embedded domain-specific language with JavaScript as the host language. CBL is implemented as a JavaScript library and any JavaScript code can be used within CBL scripts. However, CBL is designed in such a way that pre-existing knowledge of JavaScript is not necessarily required in order to write useful CBL scripts. Since CBL is valid JavaScript, it can run in most web browsers. In the following we will give an overview of CBL syntax and semantics.

### A.1 Syntax

CBL syntax is similar to JavaScript syntax. A detailed overview of JavaScript syntax can be found for example in Flanagan and Ferguson (2011). CBL differentiates between global methods, called “CBL methods” and local methods, so called “Script methods”. CBL methods allow developers to name, start and run scripts, that determine chatbot behavior with script methods. An overview of CBL methods and script methods and their respective meaning is given in Appendix A.2 Examples of syntax of CBL are given in Code Examples 1 and 2.

### A.2 Semantics

Here we describe methods specific to CBL, how they are used and what effect they have. Importantly, the listed methods are not a comprehensive overview of methods that can be used within CBL, as CBL accepts any JavaScript method. Methods that are listed here, are specifically designed for simulating chatbots with CBL in order to make chatbot simulation and data collection of simulated chatbots easier. We differentiate between CBL methods and script methods. CBL methods are global and script methods are local methods.

#### A.2.1 CBL methods

CBL methods can be thought of as the backbone of designing experiments with CBL. They allow researchers to define the temporal succession of what users see during an experiment, e.g. instructions, followed by a chatbot, followed by a questionnaire. Moreover, CBL methods enable definition and randomization of conditions. In addition, CBL methods allow researchers to define variables that should be recorded and saved. For example, researchers may want to record which condition(s) a participant was assigned to, or which response a user gave at a particular turn of the dialogue. This is possible with CBL methods. Finally, CBL methods permit control of audio files. For example, an experimenter may be interested in how users control music playback with CUI. With CBL, music files can be played back and stopped upon user request, like “Play music” or “Stop music”. Note that any kind of audio file can be thus controlled and CBL methods are not limited to music playback. In Table 3 we list CBL methods and describe how they can be used.

**Table 3:** CBL methods with descriptions of their workings and examples of usage

Method	Description and Examples
cbl.script(”scriptname”, fun)	This method defines a CBL chatbot script. Within the function, script methods – as described in the next section – are used.
cbl.instructions(fun)	This method defines an instruction page. Within the function HTML is emitted which describes the layout and content of the instructions. Multiple instructions pages can be defined by using this method multiple times.
cbl.survey(”surveyname”, fun)	This method defines a survey script. Within the function, survey methods – as described in a later section – are used.
cbl.start(”scriptname”)	This method starts the chatbot interaction that is defined by cbl.script. If any instructions are defined they will be displayed first. This method should only be called once, usually at the end of the script.
cbl.run(”scriptname”)	This method lets you switch to another script. No instructions are displayed.
cbl.completed(fun)	This method defines a page that communicates to participants that they completed the experiment.
cbl.random_item([a, b, c, ...])	This method returns a random item from an array. This can be used for automatically assigning experimental conditions to participants.
cbl.random_num(min, max)	This method returns a random number between two numbers min and max. The interval defined by min and max is inclusive. This method is another way to randomly assign conditions, in this case numbered conditions.
cbl.set(”varname”, value)	This method sets a system variable to a value. For example, the system variable “audio_prefix” is used to determine where to search for audio files. The variable “audio_prefix” can be set to search for the audio files locally under a specified path or on a publicially accessible server specified by an URL.
cbl.set_result(”colname”, value)	This method sets a survey result column to a value. This can be used to collect behavioral data in runtime, e.g. when a user puts in a specified keyword, for example “Yes” or “No”, this can be logged in runtime as a value in a column of the dataset.
cbl.play_audio(”filename”)	This method starts playback of an audio file if that audio file can be accessed via the path specified in cbl.set.
cbl.stop_audio()	This method stops playback of an audio file.

#### A.2.2 Script Methods

In CBL, scripts can be thought of as detailed implementations of dialogues. While CBL methods control the flow of the experiment, script methods control the flow within a dialogue. That is, script methods allow experimenters to define a dialogue flow for a chatbot interaction.

From a programming perspective, script methods do not exist outside the context of a given script. Scripts are objects and script methods are methods of that object. In Table 4 we list script methods used within CBL and describe their usage.

**Table 4:** Script methods with descriptions of their workings and examples of usage

Method	Description and Examples
s.begin(fun)	This method executes a function (e.g. the chatbot saying “Hi!”) when the script begins. This is in contrast to the methods s.match and s.match_if that expect a user input before executing a function.
s.match(str|regexp, fun)	This method executes a function when the user input matches the defined string or regular expression. An example JavaScript regular expression is /hello/i which will match the word hello regardless of case (hello, Hello, HeLLO, etc.)
s.match_if(str|regexp, expr_fun, fun)	This method conditionally executes a function when user input matches the defined string or regular expression and expr_fun returns true. This gives dialogue designers additional control over chatbot behavior. For example, a chatbot designer may want a chatbot to react in one way when a user says “Yes” and they are in the control condition, than when a user says “Yes” and they are in the experimental condition. This is possible with expr_fun.
s.unknown(fun)	This method executes a function when the user input is not matched. This is an important method for dialogue design, as it is impossible to predict everything users may say. Hence experimental design should account for unexpected user behavior, which is possible with this method.
s.do(”text”)	This method simulates receiving user input and attempts to match the simulated input with the s.match or s.match_if definitions. You can think of s.do as a housekeeping method that allows researchers to write code with less redundancies by letting them “jump” to sections code defined by match methods. The example scripts conditions.js, gender-modes.js and gender-subscript.js in the CBL repository showcase usage of s.do.
s.sub(”subname”, fun)	This method defines a subscript, a child script with it’s own context.
s.ret()	This method returns from a subscript to the parent script without executing the function defined in s.begin again.
s.run(”subname”, args)	This method runs a subscript and is useful for implementing turn-taking in the chatbot interaction. There are example scripts in the CBL installation folder showcasing use of this method.
s.set(”varname”, value)	This method sets a script variable to a value.
s.play_voice(”voice”, ”text”, opts)	This method speaks text defined in the method. For this the method using either browser-specific speech synthesis functions or pre-recorded audio.
s.get(”varname”)	This method gets the value of a script variable. This can be used to refer to a previously set variable, e.g. the condition a participant is assigned to.
s.say(”text”, opts)	This method sends a message to the user. If the text is an array of strings then one string is randomly selected. The voice can be supplied in the options, for example s.say(”hello”, {”voice”: ”Mary”}). The text of the message is also spoken.
s.pause(ms)	This method pauses the script for the defined number ms of milliseconds before saying more text. This can be used to assess user experiences and behavior depending on different wait times.
s.ready(fun)	This method executes a function when all pending say and pause commands are completed.
s.restart()	This method restarts the current script. An example of an application is given in the CBL repository in the example script conditions.js.

#### A.2.3 Survey methods

In CBL, survey scripts can be thought of as detailed implementations of questionnaires. Survey methods define the presentation of the questionnaire, by determining questionnaire sections, questionnaire items and scales (e.g. semantic differential scale, Likert scale, drop-down menu or input range).

In Table 5 we list script methods used within CBL and describe their usage.

**Table 5:** Survey methods with descriptions of their workings and examples of usage

Method	Description and Examples
s.section(”text”)	This method structures the layout of the survey by vertical spacing and shows users a title or instruction for filling out a section within the survey, which is defined in ”text”.
s.select(”colname”, ”text”, list, opts)	This method generates a drop-down menu, with items defined in list. The variable ”colname” defines the name of the column in the dataset, the user selection is saved under. The variable ”text” determines text shown next to the dropdown menu, e.g. a question like “What is your gender?”. The variable ”opts” defines layout aspects of the drop-down menu, like it’s width.
s.textarea(”colname”, ”text”, opts)	This method generates a free text entry box for multiple lines of text.
s.input_text(”colname”, ”text”, opts)	This method generates a free text entry box for a single line of text.
s.input_range(”colname”, ”text”, min, max, opts)	This method generates a text entry box for numeric values that accepts entries in a determined range. This is useful when asking for age. The variables are analogous to the method s.select. The variables min and max set the minimum and maximum values of the accepted range respectively. The values are inclusive.
s.sem_diff_scale(list, opts)	This method generates a semantic differential scale, useful for presenting questionnaires like AttrakDiff (Hassenzahl, Burmester, & Koller, 2003) or UEQS (Schrepp, Hinderks, & Thomaschewski, 2014). The list variable is a list of lists. Each item in list is constructed as follows: [”colname”, ”left word”, ”right word”]. The ”left word” represents the left word of the semantic differential and the ”right word” describes the right word. The variable opts determines the scale range, e.g. 7 selection options between left and right word.
s.likert_scale(list, opts)	This method generates a Likert scale, useful for presenting questionnaires like System Usability Scale (SUS) (Brooke, 1996). The variables work analogous to what is described for s.sem_diff_scale with the exception of list items that are constructed as follows: [”colname”, ”text”], with the ”colname” determining the column name of the item and ”text” depicting the text of the item, e.g. ”I feel very confident using the system”.

## Appendix B: Example study with CBL

We used CBL to set up an example experiment. Therefore, dialogue flows were designed to test different dialogue strategies that allow to ask for personal information while respecting users’ privacy. The dialogue trees are shown in Appendix B.1. We investigated users perceptions using the survey shown in Appendix B.2.

### B.1 Dialogue trees

We show dialogue trees for the example experiment for both scenarios and conditions. In Figs. 5 and 6 we visualize the dialogue flow for the banking scenario for the enter and access condition. In Figs. 7 and 8 we show dialogue trees for the location scenario for the both conditions.

### B.2 Survey

Appendix B.2 shows the questionnaire items used in the example experiment. We show the items together with the corresponding construct they were supposed to measure. Those were not presented to the participants as it might have influenced their responses. The three screening questions which are here shown after one another were distributed over the questionnaire.

## Questionnaire used in the example experiment

### Screening Questions

- **1a.** It is important that you pay attention to the statements. Please agree by choosing ‘strongly agree’ from the options.
- **1b.** To ensure that you are paying attention, please select ‘strongly disagree’ from the options
- **1c.** I recognize the importance of paying attention to the questions in the questionnaire. Please select ‘strongly agree’ to confirm your agreement.

### Frustration

Indicate to what extent you have felt this way while interacting with the chatbot

- **2a.** **frustrated**

### Privacy perception

Indicate to what extent you agree with the following statements.

- **3a.** I think this chatbot shows concern for the privacy of its users
- **3b.** I feel safe when I send personal information to this chatbot
- **3c.** I think this chatbot abides by personal data protection laws
- **3d.** I think this chatbot only collects user personal data that are necessary for its activity
- **3e.** I think this chatbot respects the user’s rights when obtaining personal information
- **3f.** I think that this chatbot will not provide my personal information to other companies without my consent

### Usability

- **4a.** With this chatbot everything is easy to understand
- **4b.** This chatbot is simple to use, even when using it for the first time
- **4c.** It is easy to find the information I need from this chatbot
- **4d.** The structure and contents of this chatbot are easy to understand
- **4e.** It is easy to move within this chatbot
- **4f.** When I am using the chatbot I feel I am in control of what I can do
- **4g.** I would like to use the chatbot frequently

### Questions about you:

- **5.** Gender
  ◦: Male
  ◦: Female
  ◦: Diverse
  ◦: I prefer not to say
- **6.** Age:
- **7.** Are you a native English Speaker?
  ◦: Yes
  ◦: No
- **8.** How often do you use chatbots on average?
  ◦: Not at all
  ◦: Less than once a month
  ◦: 2-4 times a month
  ◦: more than once a week
- **9.** You can leave comments here (optional):

Thank you for your participation in our research! If you did not enter personal information during the interaction, no personal data of yours was accessed.
